# The Role of Algorithms in Molecular Tumour Boards—Managing the Gap Between Research and Clinic in Precision Medicine

**DOI:** 10.1111/1467-9566.70040

**Published:** 2025-05-02

**Authors:** Dominik Hofmann, Elena Esposito

**Affiliations:** ^1^ Faculty of Sociology Bielefeld University Bielefeld Germany; ^2^ Department of Political and Social Sciences University of Bologna Bologna Italy

**Keywords:** algorithms, molecular tumour boards, molecularization, precision medicine, translational medicine

## Abstract

The article explores the role of algorithmic procedures in the implementation of the programme of precision medicine (PM), currently pursued in molecular tumour boards (MTBs) that emerged from the confluence of previous tumour boards and the increasing molecularisation of medicine. Our observation of the deliberations in MTBs, confirmed by interviews with participants, shows that the crucial contribution of algorithms in all stages of the processing of molecular data is neither acknowledged nor mentioned. One reason, we argue, is that these highly innovative technologies are very distant from the traditional skills and training of clinicians. The mediation through MTBs provides algorithmic procedures with the viability required to be implemented in medical decisions—and is more effective the more it goes unnoticed. Contrary to the widespread assumption of a blurring boundary between research and care, we claim that the intensification of contacts and exchanges among research endeavours and clinical operations makes the separation between the two fields increasingly sharp. As a consequence, there is a need for new forms of translation, which are accomplished by MTBs.

## 1 Introduction

Almost 10 years after the launch of the Precision Medicine Initiative in the USA the emphatic promises of the beginnings have not been realised, but the underlying project has gone on and expanded worldwide, with relevant consequences for the practice of medicine. As we will see below, precision medicine (PM) is now being pursued in entities that were not envisioned at the outset and do not have explicit references to precision: molecular tumour boards (MTBs) that emerged about 10 years ago (Erdmann 2015; van der Velden et al. 2017) to support clinical decision and have spread rapidly.

The basic idea of the ‘precision’ approach in medicine is to use the innovative scientific insights produced by human genome sequencing and analysis to customise clinical decisions. Extensive sociological literature on this topic deals with aspects such as ethical consequences of the increasing genetification of life (Juengst 2000; Rose 2007), implications for patients and doctor‐patient relationships (Eyal et al. 2019; Kerr and et al. 2021), the supposedly growing reductionism of medical reasoning, exaggerated expectations (Tutton 2012; Abettan 2016), the influence of non‐academic research industry (Robinson 2020), and others. We focus on a new type of organisational entities that are hailed as spearheading the ‘translation’ of precision into concrete clinical use. Molecular tumour boards are dedicated groups of experts with various competences also outside the medical fields, typically including clinical oncologists, pathologists, geneticists, pharmacologists, bioinformaticians and molecular biologists. They meet in person or virtually to evaluate complex cancer cases and provide personalised treatment recommendations for individual patients based on molecular analysis of tumours (Tsimberidou et al. 2023).

This paper, which is in the wake of numerous sociological studies on MTBs (Nelson et al. 2013; McGraw et al. 2017; Bourret and Cambrosio 2019; Cambrosio et al. 2021; Ackerman 2022), is based on our empirical investigation of the activities performed in the MTBs at two European Cancer Centres, that was guided by two questions: what is the role of MTBs in the implementation of the PM project? What does the observation of MTBs' meetings reveal about the relationship between research and care in advanced medicine?

We anticipate right away the result of our research: the workflow of MTBs is complex and very relevant for the implementation of PM, but in MTB sessions apparently relatively little happens. The meetings take place in a routine manner, there is little and often no discussion among the participants, the time devoted to each case is short and tends to get shorter, and the outcome is mostly the approval of pre‐prepared recommendations. As we will elaborate in more detail later, precisely this inconspicuous appearance of the activities in the sessions underlies the fundamental function of MTBs and the transformation taking place in the medical field. We trace the role of MTBs to the impact of algorithmic practices in medical research and the clinic.

By algorithmic practices we mean procedures based on advanced forms of machine learning and the use of large amounts of heterogeneous ‘big data’. They require a process of ‘algorithmization’ that involves converting analogue information into digital data, which is then processed by largely autonomous algorithms. This can enhance efficiency, accuracy and scalability, and yield results in many different fields that could not be achieved otherwise. It often goes hand in hand with the introduction of personalised predictions, databases, and automated decision support. In medicine this implies translating biological samples and specimen into digital patient data via next generation DNA sequencing, engaging with extensive health information platforms, discovering and interpreting advanced biomarkers via omics technologies, and calculating nonlinear statistical associations. Thus, algorithms make it possible to focus diagnosis and treatment on individual patients, enabling in practice precision medicine altogether. These highly innovative technologies are distant from the traditional skills and training of clinicians, and require complex forms of mediation to be seamlessly integrated into medical practice. MTBs—we argue—accomplish such translation.

Analysing this mediation, our study fits into the research strand on translational medicine, a branch of biomedicine that aims to bridge the gap between laboratory discoveries and clinical applications. This process involves a bidirectional approach, not only moving discoveries from ‘bench to bedside’ but also incorporating feedback from clinical practice to inform research (bedside to bench). In sociology, research on the translational aspect of recent developments in medicine tends to overlap with the debate about the transformations in the relationship between medical care and scientific research^1^ (Kraft 2013; Abettan 2016; Stoecklé et al. 2016; Boniolo 2017; Cambrosio et al. 2018; Cambrosio et al. 2021). This discussion in itself is not new,^2^ but the recent technological innovations have given it a new impetus, as they lead to an unprecedented intensification of relations and exchanges between the two fields—with the consequent need for new complex forms of translation. Many clinical results are immediately used for research, and conversely the treatment of many patients takes place within scientific trials.

This trend is generally interpreted as increasing intermingling of research and care (Cambrosio et al. 2018, 2024), a claim that circulates in varying forms and degrees. The weaker version notices that a growing number of artefacts, practices, and sites—as microarrays, randomised control trials, and MTBs (Nelson et al. 2014; Timmermans et al. 2017; Bourret and Cambrosio 2019)—are used simultaneously for research and in the clinical process. Some studies also found that this simultaneous use results in a blurring border between research and care and in the emergence of ‘hybrids’ and ‘entities sui generis’. In the strong version, this hybridisation is assumed to encompass all of precision medicine. Kerr et al. (2021, 254), for example, hold that in personalised cancer medicine ‘research can no longer be considered separate from cancer care’.

Our analysis of MTBs confirms these observations, but proposes a different *interpretation* of the blurring border and the need for translation. Compared with previous clinical decision support entities, in MTBs the relationships between research and care have multiplied and intensified: from the involvement of different expertise, to the exchange of data and tools, to the dual role of individuals participating in trials as patients and as case studies, and many others. However, for this very reason, the idea of hybridisation does not seem to effectively describe the processes underway. Precisely when research and clinical care use the same trials and the same data, as we shall see, the difference in their respective tasks and aims becomes apparent (and crucial): the clinician uses the results to treat the patient, whereas the researcher uses them to obtain results that can be generalised to other cases—as the debate in MTBs shows. Even when they collaborate, the aims, methods, and priorities of research remain different from treating individual patients. As we will argue in the discussion of our study of MTBs, the recent intensification of exchanges and contacts between research and care has not blurred their boundary, but on the contrary has made the separation between the two interdependent fields increasingly sharp and challenging. The more the complexity of the two domains increases, the more difficult it becomes to combine them. This is why translation is increasingly required—as it would not be the case if ‘all genomic research in cancer [were] a form of care, and vice versa’ (Kerr and et al. 2021, 254). There would be no separation to overcome and no translation would be needed. MTBs, instead, make possible the mutual utilisation of the work of scientific research and of clinical activity in both directions.

The article is structured as follows: we first reconstruct the emergence of MTBs from the confluence of the previous tumour boards and the trend towards molecular medicine. The following two sections present the methodology and results of our empirical observation of the concrete MTB workflow, focussing on the interrelation between technology, clinical aspects and social procedures leading to issue treatment recommendations in a German and an Italian MTB. The discussion of the findings illustrates our hypothesis about the role of MTBs in the framework of translational medicine. The conclusions reflect on this translational role with reference to the current intensification of the relationships between research and the clinic.

## 2 The Evolution of MTBs

To introduce molecular tumour boards, we start with relatively simple questions: how did the trend towards increasing medical precision lead to the establishment and proliferation of these entities? What is the relationship between molecularisation and precision and between precision and cancer treatment?

Precision medicine is usually presented as a product of the development of molecularization (de Chadarevian and Kamminga 1998). As stated by a bioinformatician we interviewed, ‘Precision medicine to me means that the group of people for whom we recommend something grows steadily smaller—and this refinement mainly takes place on the molecular level’ (Interview, 16 August 2022). In the perception of insiders, precision is connected to molecularisation. This partly explains the name of molecular tumour boards instead of precision tumour boards.^3^ The second component of the name, tumour board, points to the development of precision medicine in oncology. Over the last 150 years, developments emerging in the field of cancer research and treatment have repeatedly pioneered the wider progress in medicine (cf. Pinell 1992). MTBs are one result.

The third component of the name molecular tumour boards refers to the development of cancer centres, that appeared by the middle of the past century, gradually decoupling cancer treatment from cancer detection. Treatment underwent a tripartite differentiation in surgery, radiology and chemotherapy. Oncology emerged when these originally scattered treatment modalities were combined into specific centres with the single goal of treating cancer (Hajdu and Vadmal 2013).^4^ Within cancer centres themselves, specialisation progressed further, until it was perceived as over‐specialisation and new forums combining multidisciplinary expertise were called for. In the 1990s ‘tumour boards’ were introduced: regular meetings of specialists from various areas of expertise, discussing cases before determining a treatment procedure (Castel et al. 2012). To this day they remain a staple in cancer centres (Wright et al. 2007; Ruhrstaller et al. 2006).

In the early 2010s, pioneering research incorporated boards charged with deliberating the interpretation of genomic sequencing results (Roychowdhury et al. 2011; Dienstmann et al. 2014). The, by then, firmly established tumour board model served as a template for multidisciplinary ‘sequencing tumour boards’ (at Ann Arbour and at the Institut Gustave Roussy: Vassal 2010; Stoecklé et al. 2018). Convergence towards the name ‘molecular tumour board’ set in around 2012 and the model spread widely. Today, MTBs have been introduced in hundreds of cancer centres over the world.^5^ An MTB is a panel of experts from the different fields contributing to ‘molecular medicine’. Its aim is to allocate treatments—often, but not exclusively, ‘targeted therapies’—to cancer patients for whom baseline therapy has failed, based on the discussion of the findings yielded by the analysis of various molecular biomarkers of the patient.

Thus, from the perspective of medical practice it is entirely plausible that the emergence of MTBs, though based on advanced algorithmic procedures, are presented as the addition of new biomedical findings and diagnostic tests to already existing tools. On the cancer centres' webpages, molecular tumour boards are added to a list of interdisciplinary tumour boards besides breast cancer tumour boards, lung cancer tumour boards, etc. Continuity is emphasised and the innovative potential is ascribed to molecularisation. However, from the perspective of the evolution of scientific research, a discontinuity can be observed in the growing relevance of algorithmic practices and their unprecedented impact on treatment and patient care. In the following pages we explore this aspect and connect it with the translational role of MTBs.

## 3 Materials and Methods

To assess the role of MTBs in precision medicine, we draw on three different types of data. Firstly, we carried out empirical research in the MTBs of a major German and a major Italian cancer centre, complemented by observations in a virtual MTB hosted as a webinar for educational purposes and for the interchange between professionals. The webinars were a valuable data source because their setup forced participants to make occasional reflexions on what they were doing and why. We attended 17 MTB meetings at cancer centres and eight webinars over the course of 11 months from February 2022, taking notes during the sessions, and collected presentation slides. We obtained permission to register as external attendees after contacting the persons in charge of the MTBs and explaining both the objectives of our research and the guarantees of anonymity and data protection. On the basis of the recorded data, we analysed and compared the procedures, the types of indications used, and the recommendations issued. We explored especially which types of issues and indicators sparked objections, deliberations, and discussions in the expert panel.^6^

Secondly, to complement this information and check its generalisability, we collected 48 academic papers reporting procedures, cases, statistics (cohort studies) and overall experiences of MTBs all over the world (hereinafter referred to as ‘MTB papers’) based on manual PubMed searches and aiming at generating as complete a repository as possible. Nine of these papers were meta‐studies/review articles. Data points regularly provided in the papers were systematised via spreadsheet to enable comparison (meeting frequency and duration, cases per session, participants, genetic tests used and percentage of patients for whom treatment recommendations were issued/followed). The chronological reading of the MTB papers, which span the whole period of the MTBs' institutional development starting from the first pilot models, allowed us to identify trends in MTB development and institutionalisation.

Thirdly, we conducted semi‐structured interviews with 10 different persons working in and around MTBs (three of them we interviewed twice): eight MTB members from oncology, pathology, molecular pathology, genetics and bioinformatics, one also serving as MTB coordinator, one MTB coordinator that is not an MTB member and one bioinformatician that is not an MTB member, but has been involved in the development of a MTB knowledge base. A first part of these interviews was conducted at the beginning of the research activities and focused on the general structures and functioning of the MTBs, their status within the field of precision medicine, as well as the interviewees' perception of their role within it. A second round of interviews complemented our ethnographic work. Here we focused on clarifying points of interest registered during the MTB sessions. Some interview partners only granted permission to be cited indirectly, not verbatim.

At no moment during our research we entered in direct contact with patients. All patient data came in anonymised form. Before attending MTB sessions we signed an obligation of secrecy. Our research was approved by our University's Ethics Committee.

## 4 Results

In this section, we present our findings following the steps of what the insiders call the ‘MTB pipeline’. To prepare our point about the ‘algorithmisation’ underlying this process that will be thoroughly discussed in the subsequent section, we run through the pipeline twice: first as a ‘workflow’, and then as a ‘data flow’.

### 4.1 The MTB Workflow

#### 4.1.1 Selecting the Cases

In current praxis, the ‘precision patients’ (Dam et al. 2022) who enter the MTB workflow are selected with specific criteria. Most of them already received ‘baseline therapy’ (radiotherapy, cytostatic and immunotherapeutic drugs, surgery or a combination thereof). In almost all German cases, eligibility for discussion in MTBs requires failed first‐line therapies, with additional criteria—for example, patients younger than a certain age or low scores on a life‐expectancy index. Often patients must be referred to MTBs by a ‘traditional’ organ tumour board.

These criteria follow the principle of administering new, not yet widely tested treatment regimens primarily to patients for whom ‘nothing more can be done’ (Löwy 1995), and simultaneously making them part of trials. As a consequence, MTBs deal mostly with ‘rare cases’, featuring uncommon combinations of traits in terms of physiology, pathology, and drug response. As ‘rare cases’, MTB patients are important objects of research in areas where little clinical information is available. At the level of care, inscription in a study is often the only way to ensure access to targeted off‐label therapies (de Vries et al. 2019).

Patients possessing eligibility can be registered for discussion by their treating physicians, whereupon samples are taken for molecular analysis (if not already existing). Until recently these samples were surgically removed tumour cells. Presently, the less intrusive ‘liquid biopsy’ is gaining ground. Several tests and procedures based on ‘next‐generation sequencing’ are run on the sample. This can refer to standard panel tests (assays testing the expression of up to 400 genes most often associated with pathogenic traits), to whole‐exome or whole‐genome sequencing. Our interviewees informed us that in cancer centres institutionally linked to university hospitals sequencing and bioinformatic analysis are usually realised by in‐house laboratories, otherwise outsourced to private sequencing providers. ‘Analysing’ the sequenced data means first scanning them for genetic alterations, then assessing their ‘actionability’. Variants are labelled as clinically ‘actionable’ when evidence of their pathogenicity coincides with existing potential therapies.

#### 4.1.2 Interpretation and Annotation of Molecular Data

The MTB‐pipeline starts when the MTB representative is notified of the availability of the test data for local download. At the sites we observed, the cases to be discussed were distributed among a team of pathologists, who prepared a report for each, based on the molecular interpretation—the ‘annotation’ of the data. The individual reports are later combined into a single digital presentation shown during the meeting, following the tradition of medical slide talks in staff meetings. In the presentations we observed, a single patient's case takes up two or three slides with the following information in standardised and much condensed form: sex and age (names removed or anonymised), anamnesis and treatment history, type of sequencing conducted, primary tumour entity and status of proliferation, a set of molecular biomarkers (mainly ‘tumour mutational burden’ and ‘microsatellite instability’). Most of the space is taken up by a list of actionable mutations, grouped according to whether they are indicative of an association with the pathology or of interaction with treatments (e.g., resistance against a certain class of drugs), sometimes complemented by a list of variants of unknown significance.^7^

Creating this list and assessing the quality of evidence for actionability is the central part of the pathologist's preparatory work for the MTB session (Interview with a pathologist, 19 October 2022). This assessment is based on information extracted from digital knowledge bases, often using software tools facilitating the aggregation of data from different sources.

The information about actionable mutations is located centrally in the report, often as a table with the most relevant ones marked by hierarchical ordering or colouring.^8^ At the end of the report there is usually a suggestion for further proceeding. As registered in our notes from the MTB sessions and specified via interview (22 November 2022), this recommendation can consist in a treatment rationale, further diagnostic testing, referral for ‘genetic counseling’ when hereditary variants were found, inclusion into a trial or the declaration that none of the former options is warranted due to lack of actionable information.

The greatly dominant type of information entering MTB meetings is a selection of patients' genetic mutations. Patient histories are reduced to small sets of abbreviations. No radiographies or other image data are presented. The original idea of an interdisciplinary meeting where all disciplines contribute their data and professional expertise gives way to a format in which the different types of expertise focus on one very particular type of data. Although encompassing several disciplines and fields of expertise, MTB are extremely specialised bodies.

#### 4.1.3 The MTB Session

The presentation containing the reports on all patients is sent in advance to participants—a team of 10–15 people (in the published MTB papers we found numbers up to 28) representing the disciplines listed above, plus the treating physician. Active communication between the three areas involved in the preparation of the MTB session, that is, pathology, treating physician and those creating the report takes place before the actual session (Interview 7 October 2022).

The proper discussion happens in the regular (often weekly) MTB session. Although the Italian MTB convened per video conference, the German one adopted a hybrid fashion, in which remotely connected participants appeared on an additional screen besides the one of the presentation. According to MTB webpages and the MTB papers, the trend towards ‘virtualised’ MTB sessions significantly increased during the COVID‐19 pandemic.

MTB processes are highly routinised and tailored to quick and effective working through the cases.^9^ In fast pace and highly technical language the single points on the slides are passed over, either by the presenting physician or by the experts responsible for the individual data points (molecular pathologists, geneticists etc.). They summarise the findings, normally without offering explanations or interpretations. In some cases, but not all, the focus goes to unusual, potentially contradictory, or complicating findings. Everyone can contribute interpretations to shape the data into a coherent picture—especially determining which mutation is most likely to be the ‘driver’ of disease development, and where the recommended treatment should be directed. Examples of issues sparking discussion in meetings we observed are: correct histological classification of a tumour; reasons for failure of baseline treatment; probability of surprising data being due to measurement errors; qualification of a particular pathological profile for inclusion into a clinical study. The greater part of the discussion is devoted to explaining away inconsistencies between data points. In the default approach silence is interpreted as approval. Silent approval is broken occasionally to ask for clarifications, to mention evidence missing in the report or cite personal experiences with similar cases, but only very rarely to challenge the recommendation prepared in advance.

In most MTBs, in an hour or so all cases have been discussed. In small MTBs the number can be as low as two, and in bigger ones it may reach up to 20. In our observations, time left was used to revise doubtful aspects or aspect of scientific interest, for example, regarding studies currently under way at the cancer centre. During the MTB session, occasionally scientific questions are pointed out, labelling as ‘interesting’ pathological aspects that deserve scientific consideration.

#### 4.1.4 Issuing a Recommendation^10^

After the session closed, the responsible pathologist puts the conclusions into official writing. We observed how one MTB coordinator in charge of creating the presentation before and the report after the meeting copy‐pasted the respective paragraphs from the former into a digital form, which became the basis for a printable document. It was then sealed in an envelope and sent to the treating physician—who consults with the patient whether or not to follow the recommendation. MTB papers and meta‐studies show that the number of cases in which the recommendation is followed varies widely depending on the institutional and administrative context (ranging between 17% and 68%). Numbers are significantly higher for in‐label therapies, whereas for off‐label therapies they were initially very low and slowly grew from there on (Frost et al. 2022).

One might think of MTBs as sites for expert discussion of complex patient cases, but actually the review of the single cases tends to be short and MTB sessions do not usually comprise an extensive negotiation.^11^ Rather, they provide a forum in which pre‐prepared recommendations can occasionally be questioned or modified. In most cases (about two thirds of the ones we observed), the recommendation is approved without much ado. In an information sheet on MTBs in German University hospitals in 2022, the prototypical sessions are described as follows: ‘In the following 112 min, diagnoses, therapeutic measures and the results of genome analyses of the [33] individual patients are discussed by the experts present in just a few minutes. This is only possible within such a tight time frame because each case was extremely well prepared in advance’ (p. 14). Along with MTBs' growing institutionalisation and routinisation, also producing many protocols and pre‐established scoring systems that guide decisions, the number of cases discussed per session slowly but steadily rose. Nevertheless, for many patients still no recommendation can be issued.

### 4.2 The MTB Data Flow

From the clinical point of view, the task of the MTB ends here. However, the MTB workflow as a whole has not come to an end yet. What follows is the data upload into different databases that will be used for different ‘exploratory’ research projects. Therefore, from the scientific point of view, the activity of MTBs can also be observed from a different perspective: not as a *flow of work* but as a *flow of digital data*. According to the bioinformaticians we interviewed and the approach of the bioinformatic literature (for example, Hamamoto et al. 2022), a remarkable feature of the activities performed in MTBs is the pervasiveness of digitisation, or ‘algorithmisation’. The role of algorithms is central to most phases, much more than in common medical practices, and also compared with the activity of previous tumour boards.

#### 4.2.1 Algorithms in Data Acquisition

Producing a patient's genetic test results implies various steps, each conducted by a combination of algorithms of different complexity and entirely inconceivable without these tools (Stevens 2013). Sequence snippets must be ‘read out’, then compared and aligned to compose the larger exomic/genomic sequence. Subsequently, variations from the ‘reference genome’ are ‘called’ and classified as to their pathogenic effects, employing again a wide range of algorithms (Chang et al. 2022). The output comes in a standard format encompassing not only information about the location of a certain sequence in the genome but also comparative measures of its frequency in digital catalogues. Several algorithmically calculated scores predict the likelihood of its pathogenicity (Gomes and Ashley 2023, 2456f). This concatenation of algorithmic applications means that the patient data arriving on the molecular pathologist's computer have already gone through a long pipeline of preparatory and selective stages giving them a standardised format and order.

#### 4.2.2 Algorithmic Knowledge Bases for Data Annotation

In the preparation of the patient profiles presented during the MTB meeting, databases are the central tool. They provide data about the correlations between known variants and specific phenotypical disease traits, drug interactions, and existing scientific evidence supporting this information, partially stemming from algorithmic prediction. To collect and curate all such data, thousands of scientific papers have been searched and curated, often via algorithmic text mining. Meta‐databases and knowledge bases help query various databases simultaneously and provide visualisations of the genetic information. The data presented on the MTB slides are retrieved from such knowledge bases (in our observation VarSome and CIViC), partly automatically, partly through ‘manually run’ searches. This stage is a decisive transitory step between biomedical knowledge produced by clinical research and application for a concrete case.

In short: almost everything about precision medicine is algorithmic.^12^ Whenever a treatment recommendation is issued, hundreds of algorithmic operations have been combined to give way to it.

Although the role of knowledge bases and the algorithmic grounding of molecular medicine are well‐known and well‐researched, they are not perceived as playing a major role in MTBs.^13^ The meetings we observed occasionally discussed the ‘quality of the data’, but never its computational origin. In a webinar MTB session we attended, an oncologist arguing for a specific interpretation of patient data reflected: ‘now *I'm* using an algorithm in my own mind’ (their emphasis). MTB papers occasionally mention the commercial provider of sequencing technology, but almost never the databases that were searched. When asked directly about the role of algorithms, an MTB administrator told us that they do not play a role inside the MTB (informal conversation 18 August 2022). The technological basis of MTBs, although necessary, is seen as something external to the clinical part of PM. The discussion is focused on issues of ‘clinical utility’ (how likely is it that a given mutation is a driver in the specific patient's case; how likely that the patient will benefit from a change in treatment regimen; what trials could the patient be inscribed into), disregarding the algorithmic origin of the correlations that serve as evidence. As one of the oncologists tasked with variant annotation told us: ‘what I care about is the quality of the biomarker, not where it comes from’ (Interview 19 October 2022). After the medical approach that led to the time‐tested model of tumour boards, the role of algorithmisation is absorbed into the concept of molecularisation^14^: assessing biomedical evidence in the form of molecular biomarkers whose significance and reliability has been proven by associations found in medical studies.

One exception seems to be the attitude of bioinformaticians, whose involvement in the MTBs is praised as a central innovation, but who are the most passive and unengaged participants. The bioinformaticians we interviewed told us that MTBs are exactly the right place to observe algorithms entering the clinic (Interviews 16 August and 7 October 2022).

## 5 Discussion

Summing up the main results of our empirical research, three main patterns emerge: firstly, not much interdisciplinary discussion takes place in MTB sessions. Secondly, the genetic biomarkers being discussed are created by the application of algorithmic techniques. Thirdly, in and around MTBs algorithmisation is perceived as a part of the molecularisation of medical research, only indirectly affecting the provision of clinical care. Discussions are rare, and when they do happen they are quick and mostly uncontroversial; the number of cases dealt with tends to increase with growing institutionalisation, leaving little time for individual discussion; often the debate does not lead to a recommendation, and most of those issued cannot be followed up (cf. Frost et al. 2022).

In light of the relative hollowness of MTB sessions and of the limited reach of their services, one wonders why the model nevertheless proliferates, being praised, copied, financed and expanded globally. Our hypothesis is that MTBs as institutions play a crucial and innovative role within current medicine *although—and even: because—apparently not much happens in their sessions*. The reason is the increasing *algorithmization* of medicine: MTBs justify and control the selection process, enabled and prepared by algorithms, translating them into a form that is processable in the system of clinical medicine.

History and philosophy of science show a close connection between molecularisation and algorithmisation. They originated at the same time, together with a shift in biomedicine towards genes, proteins, and various types of sequences (Hilgartner 1995; García‐Sancho 2012; Stevens 2017). The concept of sequence lends itself particularly well to computerisation (Stevens 2013), and many of machine learning algorithms used today have been developed to solve problems that emerged in genetic research (Mackenzie 2017, chapter 7).

Although molecularisation is seamlessly integrated within the logic and rationality of the clinic, the same cannot be said for algorithmisation, which refers to processes and procedures that are fundamentally alien to the tradition of medical practice. The passage through MTBs, with their focus on molecularisation and its continuity with established clinical modalities, facilitates for physicians the adoption and use of algorithmic procedures that follow very different principles—fulfilling a fundamental function for translational medicine.

This task is multifaceted and complex, because ‘where the biomarker comes from’ actually affects the way MTBs work, distinguishing it from the activities of traditional tumour boards precisely for the groundbreaking impact of an algorithmic rationality, that is, present in every step of their operations. On the basis of the literature and of our empirical research, we observed this difference in three features that have an important impact on MTB structure and activities, and did not appear in this form in tumour boards: new mechanisms of monitoring and control, dynamic updating of exhaustive databases and issuing of precise predictions.

### 5.1 Mechanisms of Monitoring and Control

As pointed out above, although traditional tumour boards are specialised on one cancer type, MTBs deal with data potentially significant for several cancer types. This requires a different kind of control and use of expertise. Discussions in tumour boards have always been multi‐disciplinary, attempting to give all relevant fields of expertise their say, thereby monitoring the correct use of information beyond the limitations of narrow specialisation (Ruhrstaller et al. 2006). In MTBs, however, different experts give their opinion precisely *on a very narrow field of expertise* (McGraw et al. 2017). Most of the work is done in advance: the genetic variants have been selected, a treatment rationale is proposed on the basis of repositories of variants and studies, the evidence has already been hierarchically ordered. This leaves little room for outreaching contributions to the discussion of the case, partly explaining the limited amount of real debate in MTB sessions. The task of the discussion shifts towards choosing the correct combination of selections and assessing whether unusual data points are more likely to be the result of unusual genetic characteristics or are due to technical errors—and at this level there are few opportunities to make useful contributions.

At the same time, besides technical errors, new insidious forms of error emerge as a consequence of algorithmic learning. Even when algorithms work properly, their suggestions are not always correct. Research studies have found elusive problems of overfitting, bias and opacity (O’Neil 2016; and specifically for medicine: Montesinos López et al. 2022, chapter 4; Hunter and Holmes 2023, 1213ff.). Moreover, as one MTB member from bioinformatics argued, algorithms are poorly competent to give counter‐indications and find stop‐rules (interview 7 October 2022). They produce with equal ‘conviction’ relevant and irrelevant, or even nonsensical predictions. It is then critical to have an instance in charge of deciding what *not to do* with the indications of algorithms—and this requires specific expertise at different levels. The expert annotation comprises a first such level, the MTB is added as a further one.

### 5.2 Dynamic Updating of Broad Databases

One central argument backing the assumption that research and care are merged in MTBs is the double role of patients who are simultaneously trial participants. Although this pattern is far from new in medicine, its scope is. More or less *all* MTB patients figure into data sets used for precision medical research. This feature can also be explained by the algorithmisation underlying precision medicine. From the point of view of data flow and translation, MTBs simultaneously use and produce data: when MTB patients are inscribed into clinical trials and prospective studies, they benefit from access to experimental therapy and at the same time produce data for future precision medical treatment decisions.

The preparation of MTB reports heavily relies on knowledge bases and decision support systems developed by bioinformatics departments and software development companies that retrieve and aggregate from a broad variety of data‐sources information about cancer variants' pathogenicity scores, reaction to treatment, type and origin of the case‐relevant evidence.^15^ The knowledge bases are the result of a concatenation of other databases processing and interconnecting broad collections of genomic data (Cambrosio and et al. 2020), ranging from variant catalogues to specialised mutation type databases and meta‐knowledge bases. Finally, these knowledge bases are used to assign clinical significance scores to the variants discussed in MTBs.

One of the characteristics of such databases is the aspiration to broaden the scope of data collection as much as possible (Kitchin and McArdle 2016; Beaulieu and Leonelli 2021). Algorithmic databases are not fixed collections of datapoints. Each clinical application of information retrieved from a database leads to an outcome that may itself be recorded. By storing it, databases are dynamically coupled to the results of their usage (Tempini and Leonelli 2021, 8). In PM, the combination of scope and dynamic updating typical of algorithmic databases happens in MTBs, via their contribution to clinical trials. MTBs contribute on the one hand by selecting patients as participants of RCTs that test therapies, and on the other hand as a setting on their own: the added value of MTB recommendations is also evaluated via trials. The confluence of research and care in a single institution is thus not only a biomedical innovation, but also a reaction to the big data imperative.

### 5.3 Precise Predictions

A third difference between molecular and traditional tumour boards concerns the fact that MTB cases are generally further beyond ‘standard of care’ than the cases of organ tumour boards: only when the therapy that generally tends to work best did not yield any results, a subsequent search sets in. Precision is necessary where generality fails. For medicine, reacting to such failures entails the advanced forms of precise prediction that characterise recent algorithms (Rona‐Tas 2020). Only in this way PM can break with ‘trial‐and‐error medicine’.

The predictions delivered by algorithms are significantly different from those supplied by traditional statistical methods. Instead of a measure of the probability of an outcome for the populational average, they provide a single prediction for a specific case an individualised prediction (Siegel 2015). Although average effects were mostly useless for clinical medicine (Kravitz et al. 2004), the indications provided by algorithms can make it possible to target specific cases, providing concrete directions for clinical intervention. Not by coincidence the qualifications of molecular medicine as ‘precise’ and ‘personalised’ coincide with those applied to other fields in which algorithmisation takes place: from the biomedical point of view, the question of what is precise in PM can be answered by molecularisation. But medical care was always personalised. Taking into consideration the algorithmisation of precision medicine, what is now precise and personalised is primarily prediction.

The rationale of precise predictions is most visible in MTBs in the listings of predictive and pharmacogenetic biomarkers on presentation slides for the selection of treatment. Predictive biomarkers are biological indicators that predict how likely a patient is to respond to a particular treatment or therapy. Pharmacogenomic biomarker predict potential drawbacks of such an intervention (i.e., drug resistance). The combination between both has profound effects on clinical practice, changing the character of the distinction between diagnosis and therapy: as a product of the prediction of therapeutic targets, the diagnosis is no longer a form of generalised classification as it was traditionally conceived (Armstrong 2019; Cambrosio et al. 2021).

## 6 Conclusions

The algorithmic logic underlying precision medicine has some fundamental differences from the medical logic underscoring physician's traditional training, which are not captured by merely referring to molecularisation. Instead of directly integrating genomics into clinical training, MTBs introduce a new institutional body. Even if the algorithmic background is not explicitly present in MTBs' discussions, it is inherently embedded in their structure and function. Going through the MTB, biomedical evidence provided by algorithms gains the form and legitimacy it needs to be later converted into medical decisions. This translation from the computer to the bed, we hold, is the function MTBs fulfil—and is all the more effective the more it goes unnoticed.

The difference between technological advances and clinical practice reflects the disparity between research and care that always characterised the field of medicine. In carrying out their task and making their decisions, physicians refer to two distinct forms of knowledge: the constant renewal of scientific knowledge and the knowledge from their experience of interventions on the bodies of ever‐changing individual patients (Luhmann 1990, 183). Today, according to many observers, the relationships between these two kinds of competences are changing (Boniolo 2017), and the role of MTBs is being analysed accordingly (Bourret and Cambrosio 2019). Several phenomena observed in our research support this hypothesis, showing an intensification of the relationships between research and care that distinguishes MTBs from traditional tumour boards: collaboration between clinicians and researchers from a broad scope of disciplines; ‘dual role’ of the patients, chosen for reasons of treatment (nothing else to do) and of research (interesting cases); local concentration of data usage and production; adoption of digital tools in clinical practice; rapid integration of research findings into therapeutic measures and occasional switch in MTB sessions from the assessment of clinical relevance to evaluations of medical exceptionality (talk about ‘interesting aspects’; treating clinical cases as statistical cases).

Precisely on the basis of such enhancement of the relationship and exchanges between research and care, we propose a switch of perspective from the prevailing interpretation of this configuration. The intensification of the relationship between research and care is generally described as a trend towards a progressive fading of the boundary between them (Cambrosio et al. 2018), accompanied by a progressive blurring of several others distinctions in PM: between diagnosis and therapy (Cambrosio et al. 2021), prevention and intervention (Turnbull et al. 2018) and individual causality and statistical probabilism (Guchet 2016, 34f.). However, on the basis of very similar data, our analysis of MTBs suggests a different conclusion: the simultaneous use and production of scientific results in medicine does not lead to confusing the logics and structures of the two realms of research and care. It is true that technological advances produce more results that become relevant to medical practice. On the one hand, physicians need constant updates on increasingly rapid research results—they use databases, biomarkers, genetic sequencing, lists of variants, and advanced prediction techniques. On the other hand, scientific research increasingly needs large amounts of data, cases, information, and verification coming from the clinic. However, intensified relationships, do not imply intermingling—increased interdependence can also rely on sharper differentiation (Luhmann 1984, 250–279). It is only on the basis of the criteria of science that one evaluates which results are useful for research, not on the basis of their therapeutic helpfulness (as much as that may be a concern of the scientists themselves). For the clinic, an outcome only matters if it serves to cure patients, not if it serves to advance knowledge (however, much physicians may be interested in scientific progress in their role as medical researchers).

The intensification of contacts between research and care does not imply that research becomes more similar to care or can replace it, nor does it imply that care becomes more such as research—quite the opposite. The task of research is and remains to produce insights for experimentation and the increase of knowledge, not to cure patients. The task of care is and remains to treat and possibly heal the diseases of individual patients, independently of the generalisability of the results. When an innovative treatment is tested on a patient, what research uses is the data and the indications that are derived, whereas for medicine what is relevant is the state of the patient.^16^ Because of this difference, MTBs play a fundamental role in operationalising and legitimising an algorithmic logic in the clinical field. As algorithms become more sophisticated, the tasks of translation become increasingly complex and crucial. As more and more potentially relevant variables are tested with algorithmic tools and more and more potentially helpful targeted drugs enter the market, the need for procedures mediating and controlling their use in medical settings increases. This is in our view the task of MTBs, which perform not only one, but various types of translation. Their transfer of insights from precision medical analysis and research into concrete clinical use not only bridges the divide between large‐scale scientific endeavours and concrete individual patient care, but also helps sooth the introduction of new techniques and practices into the continuity of already established medical practices.

However, in this as in all other cases, translation does not imply acceptance. The actual rate of implementation of MTB recommendations is low and the reluctance to using newer, more automated (meta‐)knowledge bases, for example, shows the resistance of clinical medicine to the adoption of algorithmic tools. Further research will be needed to explore what forms this innovative configuration of medical practice takes, what difficulties it encounters and what conceptual tools can foster effective collaboration with the still distinct field of scientific research.

## Author Contributions

**Dominik Hofmann:** conceptualization (lead), investigation (lead), writing – original draft (lead), writing – review and editing (supporting). **Elena Esposito:** conceptualization (supporting), funding acquisition (lead), project administration (lead), supervision (lead), writing – original draft (supporting), writing – review and editing (lead).

## Data Availability

The data that support the findings of this study are available from the corresponding author upon reasonable request.

## Endnotes

^1^
In the literature about ‘translational medicine’, the separation between ‘research’ and ‘clinic’, usually separates conceptual and laboratory work from everything that implies contact with patients, while the sociological literature on ‘fading boundaries’ tends to refer to ‘research’ and ‘care’, where both can take place in the clinic. In this paper we adhere to this second meaning.

^2^
Keating and Cambrosio (2004, 365) hold that in biomedicine ‘biology and medicine have come together, both institutionally and intellectually, to create a hybrid practice’.

^3^
As they occasionally were called (cf. McGraw et al. 2017).

^4^
The ‘one‐size fits all’‐treatment of cancer was hailed at the time as progress (Fujimura 1988). Specialisation presupposed unification.

^5^
Due to lack of official registers and variable definitions, an exact number is difficult to estimate.

^6^
Similar to Castel et al. (2012) in their analysis of traditional Tumor Boards.

^7^
A ‘variant of unknown significance’ (VUS) is a genomic alteration whose status as benign or pathogenic is not standardly established due to a lack of population statistical data. Evaluating this status is one of the tasks of advanced machine learning algorithms.

^8^
The table's other columns indicate: drugs that potentially target the affected pathway; evidence levels assigned to the association; keys identifying existing studies; variant allele frequency; and typical histology.

^9^
Schapranow et al. (2023) report an average discussion time of 4:04 min per case. See also Tsimberidou et al. (2023, 844).

^10^
The final resolutions are labelled ‘recommendations’, not ‘decisions’, because in most cases MTBs are not legally allowed to take clinical decisions.

^11^
Somewhat at odds with the description of the discussions in a ‘genetic data board’ in Timmermans et al. (2017). The board they observed, however, was not properly an MTB, being exclusively focused on the evaluation of variants of unknown significance.

^12^
We use the terms ‘algorithmic’ and ‘algorithmization’ in the broad sense in which they have been taking hold in the public debate for the past few years, referring to (the spread of) a wide array of digital applications for extracting patterns and information from large datasets.

^13^
Indeed, knowledge bases are often regarded as alternatives to MTBs (Cambrosio and et al. 2020, 323).

^14^
Stoecklé et al. (2018) make a similar point.

^15^
Presently, the information aggregation is still often done ‘manually’ (Interview with a bioinformatician, 16 August 2022). But the term ‘manually’ refers to computational search operations on biomolecular databases.

^16^
The scientific part takes precedence ‘upstream’, when trials are chosen and designed. When clinical considerations suggest a modification of the protocol during the process, this usually leads to conflict (Nelson et al. 2014; Cambrosio et al. 2024). A new slot in a trial arm will not be created only so a patient can get off‐label treatment.
